# Delivery of Metabolically Neuroactive Probiotics to the Human Gut

**DOI:** 10.3390/ijms22179122

**Published:** 2021-08-24

**Authors:** Peter A. Bron, Marta Catalayud, Massimo Marzorati, Marco Pane, Ece Kartal, Raja Dhir, Gregor Reid

**Affiliations:** 1 Seed Health, 2100 Abbot Kinney Blvd Suite G Venice, Los Angeles, CA 90291, USA; peter.bron@seed.com (P.A.B.); raja@seed.com (R.D.); 2 ProDigest BV, Technologiepark-Zwijnaarde 94, 9052 Gent, Belgium; marta.calatayudarroyo@ugent.be (M.C.); massimo.marzorati@prodigest.eu (M.M.); 3 Probioticial, Via Enrico Mattei 3, 28100 Novara, Italy; m.pane@probiotical.com; 4 Faculty of Medicine and Heidelberg University Hospital, Institute of Computational Biomedicine, Heidelberg University, Im Neuenheimer Feld 672, 69120 Heidelberg, Germany; ece.kartal@embl.de; 5 Centre for Human Microbiome and Probiotic Research, Lawson Health Research Institute, 268 Grosvenor Street, London, ON N6A 4V2, Canada; 6 Departments of Microbiology and Immunology, and Surgery, Western University, London, ON N6A 3K7, Canada

**Keywords:** probiotic, intestinal delivery, genome analysis, vitamin B12, gut-brain health

## Abstract

The human microbiome is a rich factory for metabolite production and emerging data has led to the concept that orally administered microbial strains can synthesize metabolites with neuroactive potential. Recent research from *ex vivo* and murine models suggests translational potential for microbes to regulate anxiety and depression through the gut-brain axis. However, so far, less emphasis has been placed on the selection of specific microbial strains known to produce the required key metabolites and the formulation in which microbial compositions are delivered to the gut. Here, we describe a double-capsule technology to deliver high numbers of metabolically active cells derived from the 24-strain probiotic product SH-DS01 to the gastrointestinal tract, including the small intestine, where immune responses and adsorption of metabolites into the bloodstream occur. Based on its genome sequence, *Limosilactobacillus reuteri* SD-LRE2-IT was predicted to have the genetic capacity to de novo produce a specific metabolite of interest to brain health, vitamin B12, which could be confirmed *in vitro*. Taken together, our data conceptualizes the importance of rationally defined microbial strain characterization based on genomics and metabolomics data, combined with carefully designed capsule technology for delivery of live cells and concomitant functionality in and beyond the gut ecosystem.

## 1. Introduction

The health of the brain has become a major focus of research in the past two decades. This has been precipitated by increased recognition of brain injury, post-traumatic stress disorder, depression, Alzheimer’s disease, Parkinson’s disease and dementia [1]. Advances have been made in drug therapy, but high throughput sequencing methods identifying the impact of the human gut microbiome and the influence that beneficial bacteria can have on the brain have fueled the development of probiotic therapies. Probiotics are defined as “live microorganisms that, when administered in adequate amounts, confer a health benefit on the host” [2]. Although their impact on epithelial barrier integrity and immune status in the gastrointestinal tract is clearly established, probiotics have emerged with mostly incomplete claims of alleviating anxiety, depression, autism and improving memory.

Currently, the gut-brain axis concept is mostly based on animal data [3] and review papers [4]. Notably, some human studies have recently been performed, including four random controlled trials (RCTs) with schizophrenic patients, five RCTs on patients with depression and one RCT on patients with an anxiety disorder. Promising results were obtained for probiotics in the improvement of depression and anxiety but not schizophrenia symptoms. However, results from these pilot studies require repetition and more rigor on the duration of treatment and dosage, which have not been thoroughly investigated and deserve more attention [5]. Of the promising probiotic strains employed in human studies, little attention has been paid to selection of optimal (combinations of) strains, the metabolites they can potentially produce in the gut that could influence brain health, and the mechanism that would allow strain delivery to the small intestine. This is important given that the small intestine is the site of a substantial portion of immune interactions that probiotic strains can trigger in the host, some of which are relevant to the brain [6]. Moreover, the small intestine is the prime location where a functional epithelium permits or blocks molecules from absorbing into the bloodstream [7]. Since bacterial numbers of the small intestinal resident microbiota are relatively low compared to the colon, consumption of fermented foods or probiotic products is likely to lead to temporary dominance of the ingested species, especially in the duodenum [8]. Indeed, studies in healthy human volunteers have established distinct duodenum transcriptome responses upon encountering a probiotic strain [9]. Notably, responses to live cells are typically more pronounced compared to heat-killed control arms [10].

Examples of molecules produced by microbes in the gut that contribute to the developing and functioning brain include vitamin B9 (folate) and vitamin B12 [11,12]. One study of elderly subjects with mild cognitive impairment revealed that six months treatment with oral folic acid plus vitamin B12 improved cognition and reduced inflammatory cytokines [13]. Low blood levels of folate and vitamin B12 are also reported in Alzheimer’s disease patients [14]. Hence, there is consistent agreement that folate and vitamin B12 are key to adult and child brain health, in part by re-methylating homocysteine. This also translates to cancer, with folate limiting the aggressiveness of gliomas through remethylating genes such as platelet-derived growth factor-B [15]. An advantage of administering probiotic bacteria that, besides other prophylactic health-promoting capacities, produce folate and vitamin B12, is their capacity to continuously deliver these molecules to the small intestine through active metabolism. However, only specific *Limosilactobacillus* (formerly *Lactobacillus*) *reuteri* strains produce vitamin B12 [16,17,18], and likewise not all lactobacilli and *Bifidobacterium* strains produce folate, reiterating the importance of multi-omics driven strain selection.

After the conditions for appropriate strain selection are met, encapsulation is another pivotal factor for bacterial survival in the low-pH environment of the stomach and upon exposure to bile salts and digestive enzymes in the duodenum [19]. A number of methods, such as microencapsulation, have been developed to help probiotic cells resist stomach acid and bile [20,21], whereas a recent study also reported that the bioaccessibility of encapsulated probiotics consumed in yoghurt is much improved [22]. When several *Bifidobacterium* species and lactobacilli were encapsulated in a gastro-protective coating, highly comparable kinetics of intestinal colonization were observed compared to a five times higher dose of uncoated cells [21]. Hence, the microencapsulation technology used in these studies is a valid approach to significantly improve the survival of strains during gastroduodenal transit.

The aim of the present study was to demonstrate the importance of appropriate encapsulation for the ability of the 24 probiotic strains in SH-DS01 to retain high viable count and metabolic activity in a simulated small intestinal environment [23], enabling these cells to produce key metabolites such as B vitamins with potential to impact health through the gut-brain axis.

## 2. Results

### 2.1. Capsule Design & Delivery of Metabolically Active Probiotic Cells to the Intestine

The ViaCap^®^ dual capsule system employed here consists of an outer and inner swallowable capsule for enteral administration of a synbiotic composition. Both the inner and outer capsules are composed of varying thickness of hydroxypropyl methylcellulose, which is manufactured from cellulose derived from pine and spruce trees. The outer capsule is pigmented with copper chlorophyllin and is composed of prebiotic punicalagin concentrate, whereas the inner capsule contains the probiotic strains. The capsules are devoid of any agent or treatment that alters or delays the release profile kinetics e.g., Eudragit^®^ (polymerized methacrylate). The capsule is configured to be dissolved after three hours in the environment of the human stomach and small intestine which were investigated here. We assessed the bacterial release profiles of ViaCap^®^ in a modified version of the SHIME^®^ gut model that mimics the stomach and small intestine [24]. Moreover, flow cytometry–based profiles of live and dead fractions of the cells were generated.

**Figure 1:**
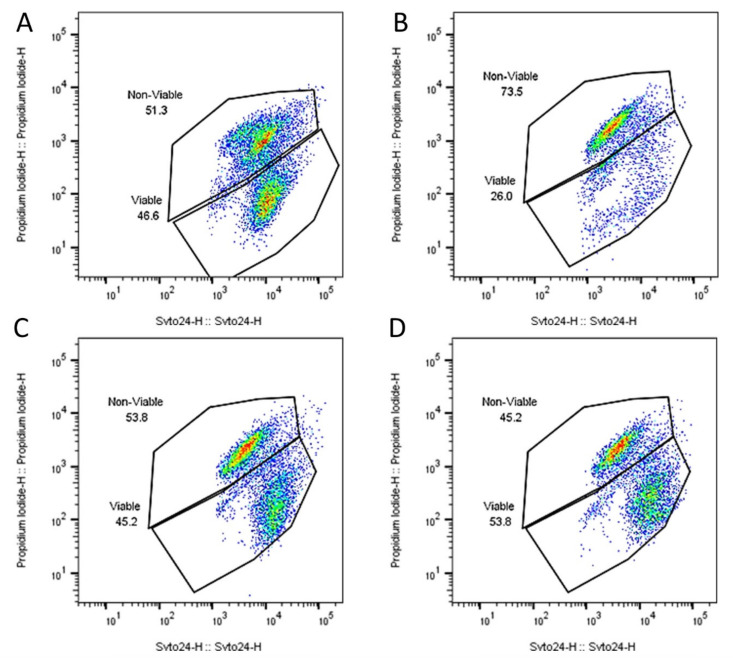
Representative flow cytometry dot plots of viable and non-viable probiotic cells in the SH-DS01 capsule (**A**) at the end of the gastric digestion stage (**B**), after 1.5 h of small intestinal digestion (**C**) and at the end of small intestinal digestion (3 h) (**D**). Bacterial cells were quantified with a double fluorescence staining of propidium iodide (non-viable cells) and SYTO24 (viable cells).

The experiments revealed that prior to employment in the SHIME^®^ system an average of 8.1 ± 0.8 × 10^10^ freeze-dried cells were present in each inner capsule, with an average of 46 ± 0.5 per cent being alive, across the 3 replicates (Figure 1 and Table 1). The number of living cells falls within the product specifications (>4 × 10^10^) and represents a number on the higher end of what is typically employed for probiotic products in clinical trials. Visual inspection in the stomach compartment of the SHIME^®^ system revealed that the capsules had started to decompose, but the bulk mass remained inside the capsule and consequently was not exposed to stomach conditions. Concomitantly, a minor fraction of cells (average of 2.8 ± 2 × 10^9^; Table 1) was detected in the gastric compartment of the SHIME^®^ system. Of this small subset of cells exposed to the stomach conditions, a larger proportion was dead (73.4 ± 4.5%; Figure 1 and Table 1) as compared to the product capsule, showcasing the detrimental effects of the stomach on probiotic survival and the requirement for appropriate encapsulation to avoid bacterial release in the stomach.

**Figure 2:**
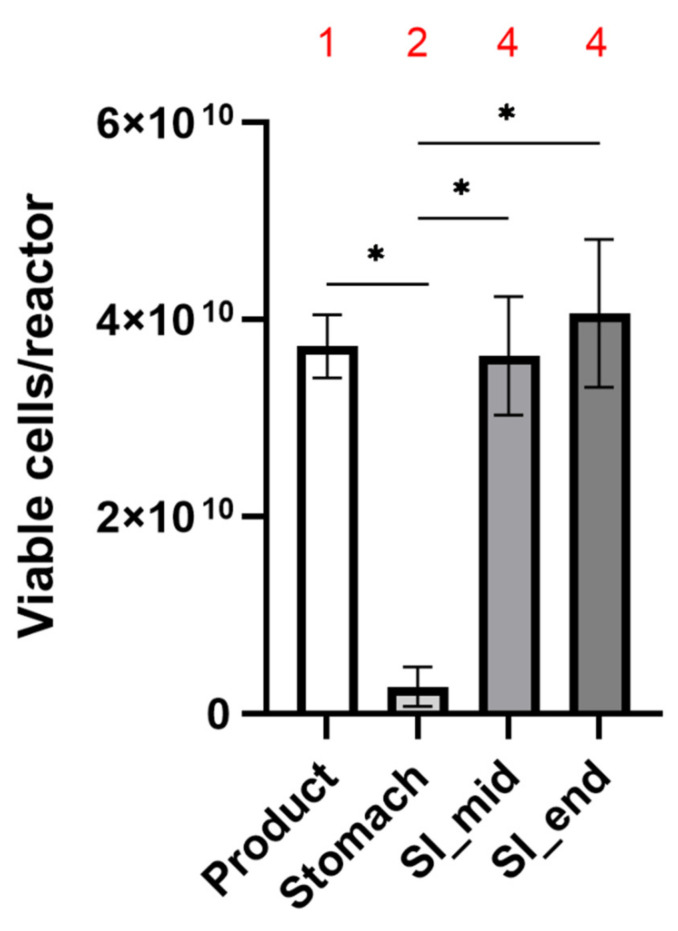
Probiotic survival during the upper gastrointestinal digestion in vitro. Bars represent the mean viable count ± SD (*n* = 3) of microbial cells obtained by double live/dead staining and flow cytometry in the product and at different gastrointestinal compartments of the simulated digestion. SI mid = conditions mimicking middle of the small intestine; SI end = conditions mimicking the end of the small intestine. Statistically significant differences in different samples are indicated with asterisks (* *p* < 0.001). The visual scores of the capsules are indicated above the bars in red: (1) capsule intact; (2) capsule starting to degrade but most bacteria retained; (4) capsule fully disintegrated.

From the upper compartment of the small intestine onwards, full disintegration of the capsule occurred, which was reflected by viable probiotic bacterial numbers (3.6 ± 0.9 × 10^10^ and 4.1 ± 0.8 × 10^10^ mid and end of small intestine, respectively; Table 1, Figure 2) highly comparable to the numbers established for the capsule prior to the experiment. Furthermore, the percentage of live bacteria (45.4 ± 8.6% and 46.2 ± 7.9% for mid and end small intestine, respectively; Figure 1 and Table 1) also mirrored that of the capsule. These data demonstrate high probiotic survival throughout the SHIME^®^ system, indicating that the segregated dual encapsulation technology permits survivability through the detrimental conditions present in the stomach.

### 2.2. Genomic & Functional Analysis of Vitamin B12 Production by L. reuteri SD-LRE2-IT

DNA isolated from *L. reuteri* strains SD-LRE2-IT and SD-RD830-FR (both present in the SH-DS01 blend) was subjected to combined Illumina and Nanopore sequencing. Both sequence data sets could be assembled into one circular contig of 2.3 (GC% 38.8 with 2424 coding sequence regions (CDS)) and 2.1 Mbp (GC% 38.9 with 2088 CDS), respectively, indicating no plasmids are present in these strains. Using average nucleotide analysis (ANI) and the publicly available genome sequence of the type strain *L. reuteri* DSM 20016 (2.0 Mbp), revealed 98.8% and 98.4% for strains SD-LRE2-IT and SD-RD830-FR, confirming species identity.

**Figure 3:**
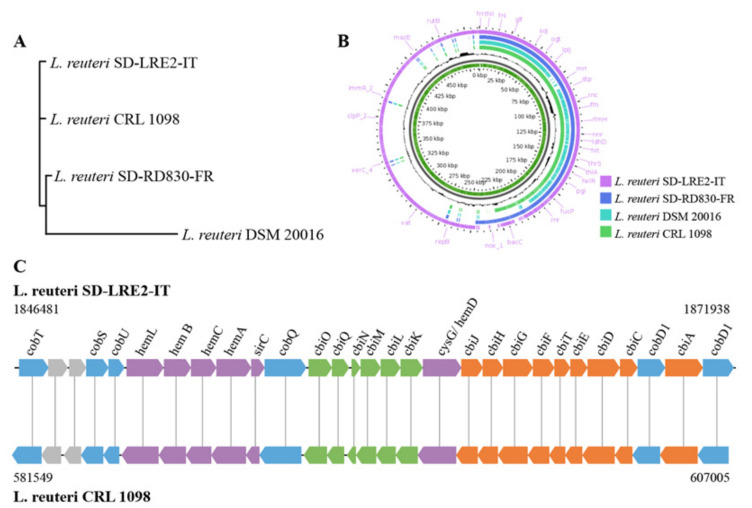
Genomic comparison of *L. reuteri* strains. (**A**) Phylogenetic trees based on the whole genome of all *L. reuteri* strains. (**B**) Pangenome analysis of four *L. reuteri* strains. The inner-most green slot shows the constructed pangenome using all four *L. reuteri* strains. The pangenome is constructed by appending unique regions onto the initial seed genome (in this case *L. reuteri* CRL 1098). The inner black slot represents the % GC content of the genomes. The other four outer slots display the BLAST hits of each strain. Empty regions on the query slots indicate areas where there were no BLAST hits between the reference *L. reuteri* CRL 1098 and the three query files (*L. reuteri* strains DSM 20016, SD-LRE2-IT and SD-RD830-FR respectively. (**C**) Schematic representation of the coenzyme B12 biosynthesis gene cluster of *L. reuteri* SD-LRE2-IT and comparison of gene order with the *L. reuteri* CRL 1098 strain [18]. The numbers 1846481 to 1871938 represent the B12 biosynthesis gene cluster of *L. reuteri* strain SD-LRE2-IT and 581549 to 607005 represent *L. reuteri* strain CRL 1098. The color code represents genes that are involved in the synthesis of: uroporphyrinogen III (purple); the lower ligand (blue); adenosylcobinamide (orange); cobalt transport (green); and not related to B12 biosynthesis when depicted in grey. Functional annotation: *cobD*, threonine-phosphate decarboxylase (EC 4.1.1.81); *cobS*, adenosylcobinamide-GDP ribazoletransferase (EC 2.7.8.26); *cobT*, *n*icotinate-nucleotidedimethylbenzimidazole phosphoribosyltransferase (EC 2.4.2.21); *cobU*, adenosylcobinamide kinase (EC 2.7.1.156)/adenosylcobinamide-phosphate guanylyltransferase (EC 2.7.7.62); *hemL*, glutamate-1-semialdehyde 2,1-aminomutase (EC 5.4.3.8); *hemB*, d-aminolaevulinic acid dehydratase (EC 4.2.1.24; hemC, porphobilinogen deaminase (EC 2.5.1.61); *hemA*, *glutamyl-tRNA reductase* (EC 1.2.1.–); *sirC*, *glutamyl-tRNA reductase* (EC 1.2.1.–); *cobQ*, cobyric acid synthase; *cbiO*, cobalt transport ATP-binding protein; cbiP, adenosylcobyric acid synthase (EC 6.3.5.10); *cbiQ*, cobalt transport protein; *cbiN*, cobalt transport protein; *cbiM*, cobalt transport protein; *cbiL*, precorrin-2 C20-methyltransferase (EC 2.1.1.130); *cbiK*, sirohydrochlorin cobaltochelatase (EC 4.99.1.3); *cysG*/*hemD*, uroporphyrin-III C-methyltransferase (EC 2.1.1.107)/uroporphyrinogen-III synthase (EC 4.2.1.75); *cbiJ*, precorrin-6X reductase (EC 1.3.1.54); *cbiH*, precorrin-3B C17- methyltransferase (EC 2.1.1.131); *cbiF*, precorrin-4 C11-methyltransferase (EC 2.1.1.133); *cbiG*, precorrin-5A C20-acyltransferase (EC 2.3.1.–); *cbiC*, precorrin-8X methylmutase (EC 5.4.1.2); *cbiD*, precorrin-5B C1-methyltransferase (EC 2.1.1.–); *cbiE*, precorrin-6Y C5,15-methyltransferase [decarboxylating] subunit CbiE (EC 2.1.1.132); *cbiT*, precorrin-6Y C5,15-methyltransferase [decarboxylating] subunit CbiT (EC 2.1.1.132); *cobD1*, cobalamin biosynthesis protein; *cbiA*; cobyrinic acid a,c-diamide synthase (EC 6.3.1.–); *cobD2*, threonine-phosphate decarboxylase (EC 4.1.1.81).

A phylogenetic tree was constructed based on the *L. reuteri* type strain, the two strains in the SH-DS01 blend, and the vitamin B12-producer *L. reuteri* CRL 1098 [18]. This revealed that strains SD-RD830-FR and SD-LRE2-IT are taxonomically more closely related to *L. reuteri* CRL 1098 than the type strain (Figure 3A,B). The genome of *L. reuteri* SD-LRE2-IT harbors the full set of genes required for de novo vitamin B12 synthesis (Figure 3C) in an identical organization that was earlier established in strain CRL 1098 [18], namely in the order *cobTSU*, *hemLBCA*, *sirC*, *cobQ*, *cbiOQNMLK*, *cysG/ hemD*, *cbiJHGFTEDC*, *cobD1*, *cbiA* and *cobD2*. By contrast, strains SD-RD830-FR and DSM 20016 appear to lack virtually all of the genes from this cluster.

**Figure 4:**
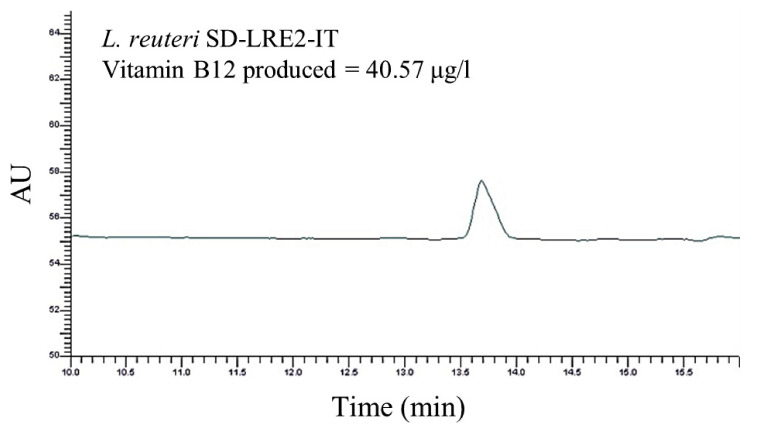
HPLC-based confirmation of vitamin B12 (cyanocobalamin) production by *L. reuteri* SD-LRE2-IT after it was grown in B12-free chemically defined medium, as outlined in 4.5. AU = arbitrary units.

To confirm the bioinformatics-based prediction of vitamin B12 production capacity of the *L. reuteri* SD-LRE2-IT, a high-performance liquid chromatography (HPLC) method was employed. The chromatographic peak for the cyanocobalamin variant of vitamin B12 appeared clearly and specifically detectable for *L. reuteri* SD-LRE2-IT at a retention time between 13 and 14 min (Figure 4), which is the anticipated retention time based on the calibration curve for which purified cyanocobalamin was employed (data not shown). This showcases the predictive power of our bioinformatics analysis for the production of a metabolite with demonstrated impacts on brain health.

## 3. Discussion

The present study employed a novel dual capsule system aiming to preserve the viability of the 24 strain SH-DS01 probiotic blend within a dry outer compartment containing polyphenolic compounds derived from pomegranate. Around half of the probiotic cells were alive within the capsule before the experiment, which is in line with earlier observations in which freeze drying survival was investigated in a wide range of species, including 84 strains of lactobacilli [25]. Viability of the lactobacilli and bifidobacteria was stably maintained throughout the intestinal model and probiotic cells were able to reach the conditions simulating the colonic environment. The delivery of the organisms at clinically relevant levels throughout the small intestine and colon is important to confer the different functionalities of the strains; for example, a large proportion of absorption of metabolites and interactions with the immune system occurs in the small intestine [8], whereas modulation of short chain fatty acid concentrations is relevant for metabolic and immune homeostasis in the colon [26]. By clinically relevant, we mean the concentration of each organism when applied earlier in clinical settings [11,27,28,29,30].

Although the concept of killed probiotics (postbiotics or parabiotics) are positioned in the market as an alternative for probiotic products, the full scale of probiotic modes of actions, notably those requiring metabolic activity, require viable organisms [31]. Hence, survivability remains a key asset which is pivotal to accurately assess products.

To utilize the predictive power of genomics we investigated the genomes of the two *L. reuteri* strains present in SH-DS01 for the genetic capacity to produce vitamin B12. This bioinformatics analysis revealed that only *L. reuteri* strain SD-LRE2-IT (DSM 23879) is able to produce vitamin B12, whereas SD-RD830-FR lacked all genetic loci involved, including the *cob*, *cbi* and *hem* clusters. Indeed *L. reuteri* SD-LRE2-IT (DSM 23879) was confirmed in vitro to have the capacity to produce vitamin B12 (40.57 ug/L). Similarly, the *B. adolescentis* strain SD-BA5-IT (DSM 18350) in the SH-DS01 blend was confirmed earlier by others to synthesize 21.92 μg of folate per billion viable cells present in 1 g of feces [11]. The excreted folic acid levels increased from 98.6 µg to 167.0 µg in 30 days, suggesting sufficient levels can be achieved for conferring health benefits related to the brain by the consumption of this probiotic strain [11]. Although only confirmed in vitro here, a similar rationale would indicate *L. reuteri* SD-LRE2-IT is capable of producing biologically relevant levels of vitamin B12. The high viability count of metabolically active cells shown to be delivered to the simulated small intestine and colon further supports this capability.

Many commercial supplements contain a range of vitamins essential for brain and other body functions. One ‘high-dose’ multi-nutrient product that included 150 ug of folic acid (vitamin B9) and 30 ug of vitamin B12 taken daily for 6 months was found to reduce homocysteine and increase brain markers for oxidative metabolism and myelination [32].

The relationship between microbial metabolites produced in the gut and mental health is a very intriguing and quickly developing field of microbiome research. In a Flemish cohort, a microbiome and metabolome approach showed that butyrate-producing *Faecalibacterium* and *Coprococcus* were consistently associated with higher quality-of-life indicators [33]. Moreover, the dopamine metabolite 3,4-dihydroxyphenylacetic acid correlates positively with mental quality-of-life, whereas microbial γ-aminobutyric acid production appears to play a role in depression [33]. Gut microbiota members have also been shown to regulate biosynthesis of the brain neurotransmitter 5-hydroxytryptamine. This modification appears indirect, as spore-forming gut bacteria modulate metabolites that in turn promote colonic 5-hydroxytryptamine biosynthesis [34]. Nevertheless, microbiota-dependent changes in 5-hydroxytryptamine impact GI motility and homeostasis and gut microbial composition alterations might improve serotonin-related disease symptoms [34].

*Akkermansia muciniphila* has been shown to ameliorate the symptoms of ALS in a Sod1-Tg mouse model. Furthermore, Sod1-Tg mice that received *A. muciniphila* were found to accumulate nicotinamide (vitamin B3) in the central nervous system, and systemic supplementation of this vitamin improved motor symptoms in the spinal cord. These results translate to humans where distinct microbiome and metabolite configurations, including reduced levels of nicotinamide, have been observed in patients with ALS as compared to healthy controls [35]. Several of the endogenous microbiota-produced metabolites linked to health status can also be produced by specific probiotic strains, including those present in SH-DS01, suggesting consumption of appropriate probiotic products could be a supportive approach for the maintenance and improvement of brain health.

Notably, probiotic strains have been shown to exert multiple health benefits through mechanisms besides metabolite production or conversion, including direct immune modulation [36] and strengthening of the epithelial barrier [37]. It would be unrealistic to expect a single probiotic strain to act through multiple mechanisms and impact the variable genetic backgrounds, microbiome composition and dietary habits of consumers. Hence, the employment of multiple strains performing different functions will likely have a higher relevance for the broader, diverse population.

## 4. Materials and Methods

### 4.1. Probiotic Product

SH-DSO1 capsules containing a 24-strain probiotic mixture, encased in a pomegranate (*Punica granatum*) outer layer, were obtained from Seed Health (Los Angeles, CA, USA). SH-DSO1 contains the following strains: *Bifidobacterium breve* HRVD521-US, *Lacticaseibacillus rhamnosus* HRVD113-US, *Bifidobacterium lactis* HRVD524-US, *Lacticaseibacillus casei* HRVD300-US, *Bifidobacterium longum* SD-BB536-JP, *Lactiplantibacillus plantarum* SD-LPLDL-UK, *Bifidobacterium animalis* ssp. *lactis* SD-MB2409-IT, *Bifidobacterium longum* SD-CECT7347-SP, *Lacticaseibacillus casei* SD-CECT9104-SP, *Bifidobacterium lactis* SD-CECT8145-SP, *Lactiplantibacillus plantarum* SD-LP1-IT, *Bifidobacterium breve* SD-BR3-IT, *Lacticaseibacillus rhamnosus* SD-LR6-IT, *Limosilactobacillus reuteri* SD-LRE2-IT, *Bifidobacterium infantis* SD-M63-JP, *Bifidobacterium longum* HRVD90b-US, *Bifidobacterium lactis* SD-BS5-IT, *Bifidobacterium lactis* SD150-BE, *Limosilactobacillus reuteri* SD-RD830-FR, *Lacticaseibacillus rhamnosus* SD-GG-BE, *Bifidobacterium adolescentis* SD-BA5-IT, *Lactobacillus crispatus* SD-LCR01-IT, *Limosilactobacillus fermentum* SD-LF8-IT and *Ligilactobacillus salivarius* SD-LS1-IT.

### 4.2. Upper GIT Model and Bacterial Release from the Capsule

A modified version of the original SHIME^®^ system [23] was employed here to represent the physiological conditions of stomach and small intestine within the same reactor [24]. In order to mimic fasting conditions, the stomach was simulated with a 60-min incubation in a simulated gastric fluid containing NaCl 5.98 g/L (Carl Roth, Belgium), type II gastric porcine mucin 4 g/L (Merck KGaA, Darmstadt, Germany), and 1 g/L pepsin (Chem Lab, Belgium) at pH 1.8. Subsequently, the small intestinal conditions were brought about by modification of the pH to 6.8, addition of pancreatin (Merck, 0.9 g/L), and an initial bile salt concentration (Oxgall with a cholic acid content of 50–70% [38]; Dickinson, Belgium) of 4.5 mmol/L, decreasing to 2.25 mmol/L in 100 min by the addition of NaHCO_3_ 3 g/L (Carl Roth, Belgium). Samples were collected for FACS analysis at the following timepoints throughout the incubation: immediately prior to testing, the end of stomach incubation (ST end), halfway through the small intestinal incubation (SI mid), and the end of the small intestinal incubation (SI end). At each sampling time point, a visual inspection of the capsules was performed to assess their disintegration behavior during passage through the different regions of the upper GIT. Different stages of disintegration were defined as (1) capsule intact; (2) capsule damaged but most bacteria in the capsule; (3) capsule damaged and most product released; (4) capsule fully disintegrated.

### 4.3. Probiotic Survivability

Quantification of viable and non-viable microbial cells was done by flow cytometry. Samples were collected at different stages of the experiment from the stomach and small intestinal compartment to determine the number of viable microbial cells by flow cytometry. A ten-fold dilution series was initially prepared in phosphate buffered saline. Assessment of the viable population of the probiotic mixture was done by staining the appropriate dilutions with SYTO 24 and propidium iodide (PI). Samples were analyzed on a BD FACSVerse (BD Biosciences, Erembodegem, Belgium). The samples were run using the high flow rate setting of the machine. Bacterial cells were separated from medium debris and signal noise by applying a threshold level of 200 on the SYTO channel. Proper parent and daughter gates were set to determine all populations. Results are reported individually and as the average (counts/reactor) ± SD of the three independent biological replicates.

Statistically significant differences between the number of viable microbial cells were determined in between each sampling point and its preceding one during the experiment to demonstrate temporal changes. In terms of statistics, the differences for all data discussed and indicated by “*p* < 0.05” or “*” were significant with a confidence interval of 95%, as demonstrated using Student’s *t*-test.

### 4.4. Sequencing and Analysis of L. reuteri Genomes for Vitamin B12 Production Capacity

Genomes were sequenced and assembled into one circular contig by a combination of Illumina and Nanopore sequencing. A 9.4.1 flow cell with the SQK-LSK109 kit was used according to the manufacturer’s protocol (Oxford Nanopore Technologies, Oxford, UK) with the following modification: removal of nicks and overhangs (DNA end prep) to create blunt ends DNA was performed by incubation of the DNA mixture at 20 degrees for 15 min and 65 degrees for 15 min. Base-calling was performed using Guppy v3.6 in high accuracy mode. For Illumina sequencing, the run was performed on the NextSeq 550 with a X2X75 PE mid-output using the DNA Nextera kit. The long-read data was assembled into contiguous DNA sequences (contigs) using the Flye assembler (version 2.8.1-b1676). Medaka (version 1.0.3) were error corrected with the assembled sequence using the long read sequences. Additionally, the contigs were further error corrected using Pilon (version 1.23) [39]. Species identity was confirmed using average nucleotide identity (ANI) analysis using the Pyani software package (version 0.2.10). Open reading frames (ORFs) of the assembled contigs were predicted using Prodigal (version 2.6.3) [40]. The genomes of *L. reuteri* SD-LRE2-IT and *L. reuteri* SD-RD830-FR were investigated for the presence of homologues of the *cbi*, *cob* and *hem* gene cluster essential for vitamin B12 production [18].

The *L. reuteri* DSM 20016 (GenBank: CP000705.1) and *L. reuteri* CRL 1098 (GenBank: LYWI00000000.1) genomes were used for comparisons. The phylogenetic relationships between strains and pangenome construction were done by the Roary pipeline [41] Genome annotations of *L. reuteri* SD-LRE2-IT, *L. reuteri* SD-RD830-FR, *L. reuteri* CRL 1098 and *L. reuteri* DSM 20016 strains were done by using prokka (version 1.14.6) [42]. Annotated assemblies in GFF3 format were used to compute pangenome constructions by Roary with default parameters of the Roary pipeline. accessed 1 August 2021. Phandango (https://jameshadfield.github.io/phandango/#/main (accessed on 1 August 2021)), GView (https://server.gview.ca/ (accessed on 1 August 2021)) and mauve (http://darlinglab.org/mauve/mauve.html (accessed on 1 August 2021)) were used for visualizations.

### 4.5. Functional Assessment of Vitamin B12 Production by L. reuteri

HPLC was employed to evaluate the production of vitamin B12 by the *L. reuteri* strains present in the probiotic product SH-DS01. Briefly, four sequential subcultures for each strain were used in a chemically defined, vitamin B12-free assay medium [18] (Himedia, Mumbai, India), allowing the complete activation of the strain and the conditioning of the growth in the culture medium devoid of vitamin B12. The strains analyzed were inoculated at the same percentage and under the same culture conditions. Subsequently, the culture broths were centrifuged and washed in phosphate buffer, followed by resuspension in an extraction buffer in the presence of potassium cyanide, a donor of the cyano- moiety required for the formation of cyanocobalamin. The suspensions were ultrasonicated in an ice bath to lyse the cells and release of vitamin B12. In order to evaluate the efficiency of the lysis event, the Bradford assay was employed and also used to obtain a comparison parameter among the various strains. Thermic treatment of the samples was then carried out to allow the definitive formation of cyanocobalamin, followed by centrifugation. Subsequently, 20 uL of the supernatant was injected into the HPLC using an Ascentis C-18 column (250 × 4.6 mm, 4 μm) and a Uv-Vis detector at 360 nm wavelength. 1.0 mL elution volume per minute was applied with a 95:5 H_2_O-acetonitril gradient. A calibration curve was generated by using a cyanocobalamin (Sigma, Tokyo, Japan) standard at different concentrations ranging from 50 ng/mL to 1000 ng/mL.

## Figures and Tables

**Table 1:** Viable, dead and total bacterial cell counts for the product at different stages of the upper gastrointestinal digestion *in vitro*. Values represent mean ± SD (*n* = 3). Product = total count in product at beginning of the experiment; ST = stomach conditions; SI mid = conditions mimicking middle of the small intestine; SI end = conditions mimicking the end of the small intestine.

	Counts			Percentage of Cells Viable		
	Viable					
Product	3.73 × 10^10^	±	3.21 × 10^9^	46.03	±	0.54
ST end	2.77 × 10^9^	±	1.98 × 10^9^	25.99	±	4.03
SI mid	3.63 × 10^10^	±	9.07 × 10^9^	45.38	±	8.56
SI end	4.07 × 10^10^	±	7.51 × 10^9^	46.15	±	7.87
	Dead					
Product	3.57 × 10^9^	±	1.78 × 10^9^	53.97	±	0.54
ST end	8.80 × 10^9^	±	7.30 × 10^9^	73.36	±	4.54
SI mid	4.30 × 10^10^	±	5.03 × 10^9^	53.57	±	8.36
SI end	4.67 × 10^10^	±	6.11 × 10^9^	53.13	±	7.79
	Total					
Product	8.12 × 10^10^	±	7.99 × 10^9^	100	±	0.5
ST end	1.16 × 10^10^	±	9.29 × 10^9^	99.35	±	0.75
SI mid	8.02 × 10^10^	±	4.36 × 10^9^	98.95	±	1.15
SI end	8.80 × 10^10^	±	2.28 × 10^9^	99.28	±	0.35

## Data Availability

The genome sequences of *L. reuteri* SD-RD830-FR and SD-LRE2-IT can be accessed at NCBI under genome submission number SUB9865138.
